# Supplementary material for: WordCommentsAnalyzer: A windows software tool for qualitative research
Source: F1000Res. 2018 Sep 4;7:536. Originally published 2018 May 3. [Version 2] doi: 10.12688/f1000research.14819.2 (PMC6137411; doi:10.12688/f1000research.14819.2)
Supplement: Supplementary file 3 [file f1000research-7-17614-s0002.tgz › b2a79496-4ada-47c4-9682-a99ce867836d.zip!/Eight Iranian Health Journals/Salmand_Iranian Journal of Ageing _22.docx]

© Salmand_Iranian Journal of Ageing . This work is licensed under CC BY-NC 4.0 (https://creativecommons.org/licenses/by-nc/4.0/).

Volume & Issue: دوره 7 - شماره 4

Title: مرور نظام‌مند مطالعات مرتبط با میزان شیوع و عوامل مؤثر بر افسردگی و مداخلات درمانی آن در سالمندان ایرانی

http://salmandj.uswr.ac.ir/article-1-545-fa.html

اهداف: افسردگی یکی از مهمترین اختلالات روانپزشکی و شایع‌ترین اختلال خلقی است. هدف این مطالعه ارائه تصویر روشنی از میزان شیوع، عوامل موثر و مداخلات افسردگی در دوره سالمندی در مطالعات ایرانی است. مواد و روش‌ها: این مطالعه از نوع مطالعات توصیفی است و با توجه به روش اجرا مرور سیستماتیک تلقی می شود. جامعه آماری کلیه مطالعاتی است که از جوانب مختلف به موضوع افسردگی سالمندان ایرانی پرداخته اند. کلید واژه‌های فارسی افسردگی،‌ دیس تایمی، ملانکولی و اختلال خلقی در: پایگاه اطلاعات علمی جهاد دانشگاهی، پژوهشگاه اطلاعات و مدارک علمی ایران، بانک اطلاعات مقالات علوم پزشکی ایران، بانک اطلاعات مقالات بهداشت روان کشور و بانک اطلاعات نشریات کشور و کلید واژه های انگلیسی ,Depression, Mood Disorder, Melancholia, Iran Dysthymia در بانک اطلاعات Medline جستجو شد. در مرحله دوم، موارد تکراری و خارج از بازه زمانی مطالعه (1375تا1390) از مطالعه خارج شد. معیار غربالگری در مرحله دوم ارتباط با موضوع مطالعه و حذف مقالات انگلیسی بود که به زبان فارسی نیز منتشر شده بودند. لازم به ذکر است که در مرحله اول غربالگری عناوین و در مرحله دوم چکیده مقالات توسط دو مرورگر مجرب بررسی، و به این ترتیب مقالات وارد مرحله ارزیابی کیفیت مطالعات گردید. ارزیابی کیفیت مطالعات بر اساس چک لیستهای ارزیابی کیفیت مطالعات موجود از جمله سایت برنامه مهارت های ارزیابی انتقادی انجام گرفت. یافته‌ها: پس از جستجو، غربالگری و ارزیابی کیفی مطالعات، در پایان سنتز نهایی بر روی 26 مقاله انجام گرفت. سنتز مقالات مرتبط با افسردگی دوران سالمندی نشان داد که میانگین شیوع این اختلال در سالمندان ساکن در سرای سالمندان با ابزار بک معادل 64/95درصد و  با جی.دی.اس 85/81درصد می باشد. همچنین شیوع افسردگی در سالمندان ساکن منزل با ابزار جی.دی.اس نیز 57/58درصد بود. از مهم‌ترین عوامل مرتبط با افسردگی دوران سالمندی در مطالعات ایرانی می توان به جنس زن،‌ تاهل، سکونت در سرای سالمندان، میزان تحصیلات، سن و وضعیت اقتصادی- اجتماعی اشاره کرد. همچنین روان درمانی،‌ دارو درمانی و ورزش بترتیب بیشترین مداخلات این گروه سنی را تشکیل می دادند.  نتیجه‌گیری: شیوع بالای افسردگی دوران سالمندی در مطالعات ایرانی، ضرورت بررسیهای بیشتر و مداخلات اصولی را با توجه به عوامل مرتبط با افسردگی این دوران طلب می کند.

Title: بررسی کارکرد بینایی و همبستگی آن با کیفیت زندگی در سالمندان استفاده‌کننده از خدمات درمانگاه‌‌‌های دولتی بوشهر

http://salmandj.uswr.ac.ir/article-1-455-fa.html

اهداف: ضعف بینایی یکی از ده علل شایع ناتوانی است. بیش از دو سوم بالغین دچار مشکلات بینایی، سالمندان بالای ۶۵ سال هستند. هدف این پژوهش تعیین رابطه کارکرد بینایی با کیفیت زندگی در سالمندان مراجعه کننده به درمانگاه‌‌‌های دولتی شهر بوشهر بود. مواد و روش‌ها: این طرح از نوع توصیفی-تحلیلی است که به روش مقطعی اجرا شده است. روش نمونه گیری تصادفی خوش‌‌های بود. برای جمع آوری داده‌ها از پرسشنامه‌‌‌های کارکردبینایی (25-VFQ) و کیفیت زندگی (36-SF) استفاده شد. داده‌ها با استفاده از ضریب همبستگی اسپیرمن، آزمون من-ویتنی وکروسکال-والیس مورد بررسی قرار گرفتند. یافته‌ها: داده‌‌‌های گرد آوری شده از ۱۷۳ آزمودنی با میانگین سنی ۶/۸±۶۹/۳ سال که ٪۷۱/۱ آن‌ها مذکر بودند مورد تحلیل آماری قرار گرفت. نتایج نشان داد که وضعیت کارکرد بینایی (۱۷/۲۴±۶۷/۸۲) و کیفیت زندگی (۲۳/۲۹±۵۶/۴۱) آزمودنی‌ها در حد متوسط بوده و رابطه مستقیم و معنی داری بین کارکرد بینایی و کیفیت زندگی سالمندان وجود دارد (P<0/0001) و این رابطه به رغم تغییر در متغیر‌‌های سن، جنس، وضعیت تاهل و سطح تحصیلات حفظ می‌شود. افزایش سن، نداشتن همسر و بیسوادی، همبستگی کارکرد بینایی با کیفیت زندگی را افزایش می‌دهد. نتیجه‌گیری: ارائه خدمات پیشگیری، غربالگری، درمان، و توانبخشی بینایی به سالمندان، خصوصاً گروه‌‌‌های در معرض خطر فوق، توصیه می‌شود

Title: تأثیر تمرین درمانی در آب بر تعادل ایستا و پویای سالمندان: مقایسه تمرینات اختلالی و غیراختلالی

http://salmandj.uswr.ac.ir/article-1-533-fa.html

اهداف: تمرینات تعادلی می تواند میزان افتادن در سالمندان را کاهش دهد. طبق اصل اختصاصی بودن تمرین، انجام برنامه‌های تمرینی اختلالی مشابه شرایط واقعی می‌تواند به نارسایی‌های حسی و حرکتی مرتبط با افزایش سن کمک کند. همچنین، استفاده از برنامه‌های تمرینی در آب می تواند محدودیت‌های اجرایی را کاهش دهد. لذا هدف از تحقیق حاضر مقایسه اثربخشی تمرینات تعادلی اختلالی و غیر اختلالی در آب بر قابلیت‌های تعادل ایستا و پویای زنان سالمند است. مواد و روش‌ها: روش تحقیق حاضر از نوع نیمه تجربی با طرح پیش آزمون، پس آزمون با گروه کنترل است. بدین منظور 37 زن سالمند با دامنه سنی 80-65 به روش نمونه گیری در دسترس انتخاب و از طریق گمارش تصادفی، به گروههای آزمایشی اختلالی (n=12)، غیراختلالی (n=12) و کنترل (n=13) تقسیم شدند. از تمامی آزمودنی‌ها قبل و پس از دوره تمرینی، با استفاده از دستگاه تعادل سنج بایودکس آزمون ثبات قامتی به‌دو صورت ایستا و پویا اجرا شد. به منظور تحلیل یافته‌ها از آزمونهای تحلیل واریانس، t همبسته و t مستقل استفاده شد. یافته‌ها: تمرین اختلالی بر همه شاخصهای تعادل ایستا و پویا بهبود معناداری ایجاد نمود (P<0/05). همچنین تمرین غیراختلالی فقط بر شاخص نوسان میانی-جانبی (ML) در هر دو نوع تعادل ایستا و پویا تاثیر معنادار نداشت (P>0/05). علاوه بر آن نتایج نشان داد تمرین اختلالی در مقایسه با تمرین غیراختلالی تاثیر بیشتری بر بهبود تمامی شاخص های تعادل ایستا و پویا داشته است (P<0/05).  نتیجه‌گیری: یافته های تحقیق حاضر، اصل اختصاصی بودن تمرین را مورد تایید قرار داد. تحقیق حاضر همخوان با برخی تحقیقات گذشته ثابت نمود تمرینات تعادلی می توانند باعث بهبود تعادل شوند، اما این گونه تمرینات برای بهبود سازگاریهای عصبی عضلانی ویژه تعادل، به حد کافی اختصاصی نیستند. بنا براین، تمرینات اختلالی می توانند پاسخهای جبرانی قامتی در اثر اختلال بیرونی را فعال کرده و در نتیجه خطر افتادن را کاهش دهند.

Title: رابطه تحرک کارکردی با تعادل و میزان وابستگی در فعالیت‌های روزمره زندگی سالمندان پس ازجراحی تعویض مفصل ران

http://salmandj.uswr.ac.ir/article-1-502-fa.html

اهداف: هدف این مطالعه تعیین رابطه همبستگی تحرک کارکردی با تعادل و میزان وابستگی در انجام فعالیت‌های روزمره زندگی در افراد سالمند پس از جراحی تعویض مفصل ران و مقایسه آن با سالمندان سالم بود. مواد و روش‌ها: در یک مطالعه بررسی-مقطعی با نمونه‌گیری به روش غیراحتمالی ساده، 15 بیمار مبتلا به استئوآرتریت پیشرفته مفصل ران با میانگین سنی 9/6±72 که 6 ماه از عمل جراحی تعویض مفصل ران آن‌ها گذشته بود، انتخاب شدند. به علاوه 15 سالمند با میانگین سنی 1/4±72/7 به عنوان گروه کنترل با توجه به همتاسازی دو گروه وارد مطالعه شدند. تحرک کارکردی با آزمون Timed Up and GO (TUG)، تعادل با آزمون Functional Reach (FR) و پرسشنامه تعادلی برگ و همچنین میزان استقلال در فعالیت‌های روزمره با پرسشنامه شاخص بارتل تغییر یافته اندازه‌گیری شد. رابطه تحرک کارکردی با تعادل و میزان وابستگی در انجام فعالیت های روزمره زندگی با آزمون آماری ضریب همبستگی پیرسون تحلیل شد. یافته‌ها: بین تحرک کارکردی با تعادل و میزان وابستگی در انجام فعالیت‌های روزمره زندگی در گروه بیمار رابطه همبستگی قوی وجود دارد (r>0/50) همچنین بین تحرک کارکردی با تعادل و میزان و ابستگی در انجام فعالیت‌های روزمره زندگی در گروه سالم رابطه همبستگی متوسط وجود دارد (r>0/30). نتیجه‌گیری: نتایج وجود رابطه همبستگی مثبت تحرک کارکردی با تعادل و میزان وابستگی در انجام فعالیت‌های روزمره زندگی در سالمندان پس از جراحی تعویض مفصل ران را نشان داد. همچنین رابطه همبستگی تحرک کارکردی با تعادل و میزان وابستگی در انجام فعالیت‌های روزمره زندگی درگروه بیمار بیشتر و بهتر از گروه سالم بود.

Title: بررسی رابطه مشارکت اجتماعی و کیفیت زندگی سالمندان عضو کانون‌های سالمندی شهر تهران در سال 1390

http://salmandj.uswr.ac.ir/article-1-655-fa.html

اهداف: هدف از مطالعه حاضر، بررسی رابطه مشارکت اجتماعی و ابعاد آن (مشارکت نهادی و مشارکت غیرنهادی) با کیفیت زندگی و ابعاد آن در میان سالمندان عضو کانون‌های سالمندی شهر تهران می باشد. مواد و روش‌ها: این مطالعه از نوع توصیفی-تحلیلی و مقطعی می باشد. جامعه آماری پژوهش شامل کلیه سالمندان عضو کانون‌های سالمندی شهر تهران می‌باشد که از میان آن‌ها، 200 نفر از سالمندان 60 سال به بالا را از میان کانون‌های سالمندی مناطق 1، 12 و 20 را به روش نمونه‌گیری خوشه‌ای و تصادفی ساده انتخاب نموده و ابزارهای پژوهش، شامل پرسشنامه کیفیت زندگی لیپاد، پرسش‌نامه محقق ساخته مشارکت اجتماعی و چک لیست اطلاعات دموگرافیک را در میان جامعه نمونه به اجرا درآورده و اطلاعات کسب شده را با استفاده از روش‌های آماری ضریب آلفای کرونباخ، ضریب همبستگی پیرسون، رگرسیون خطی و آزمون های کولموگروف-اسمیرنف، F و T مستقل مورد تجزیه و تحلیل قرار گرفتند. یافته‌ها: مقایسه میانگین نمره کیفیت زندگی (P>0/05) و مشارکت اجتماعی (P>0/05) در میان مردان و زنان سالمند تفاوت آماری معناداری را نشان نداد. همچنین ضریب همبستگی مشارکت اجتماعی و کیفیت زندگی (r=0/778) رابطه معناداری بین دو متغیر را نشان داد. با استفاده از آزمون رگرسون (Radj=0/63)، مشخص گردید که 63درصد تغرات متغیر کیفیت زندگی توسط متغیرهای این پژوهش قابل پیش بینی است. نتیجه‌گیری: نتایج پژوهش حاکی از معنادار بودن رابطه بین مشارکت اجتماعی و ابعاد آن با کیفیت زندگی سالمندان می‌باشد و مشارکت غیرنهادی در میان سالمندان غلبه بیشتری دارد.

Title: عوامل تأثیرگذار بر سبک زندگی سالم از دیدگاه سالمندان: مطالعه کیفی

http://salmandj.uswr.ac.ir/article-1-362-fa.html

اهداف: اتخاذ سبک زندگی سالم در دوران سالمندی از اهمیت قابل توجهی برخوردار است. جمعیت سالمند ایران روز به روز در حال افزایش بوده و به همین دلیل شناسایی عوامل تاثیر گذار بر ارتقای سبک زندگی آنان از دغدغه های ذهنی سیاست گذاران نظام سلامت کشورمان می باشد. بنابراین با توجه به این مهم این مطالعه به بررسی دیدگاه های سالمندان درباره عوامل تاثیر گذار بر سبک زندگی سالم در این طبقه سنی می پردازد. مواد و روش‌ها: این مطالعه به صورت کیفی و از طریق انجام مصاحبه فردی با 15 نفر از سالمندان مراجعه کننده به مرکز بهداشتی درمانی شهر شبستر انجام گردیده است. کلیه مصاحبه ‌ها ضبط و پیاده‌سازی شد و با استفاده از روش تحلیل چارچوب و برمبنای ایجاد ساختار مفهومی تحلیل گردید. یافته‌ها: سه موضوع به عنوان عوامل کلیدی تاثیرگذار بر سبک زندگی سالم سالمندان شناسایی شد. "ابعاد سبک زندگی سالم"، "حمایت اجتماعی"، "عوامل تسهیل گر در اتخاذ سبک زندگی سالم". مفاهیم توصیف کننده ابعاد سبک زندگی سالم عبارتند از: اقدامات احتیاطی، تعاملات اجتماعی، معنویت و عادات روزمره زندگی، مفاهیم توصیف کننده حمایت اجتماعی شامل خانواده، دوستان، مسئولان، رسانه بوده و مفاهیم توصیف کننده عوامل تسهیل گر در اتخاذ سبک زندگی سالم برآورد نیازهای دوران سالمندی و خودکارآمدی سالمند را شامل می گردد. نتیجه‌گیری: چگونگی اتخاذ سبک زندگی سالم در سالمندان تحت تأثیر عوامل متعددی قرار دارد. در این گروه سنی شناسایی عوامل تاثیرگذار بر سبک زندگی سالم نقش مهمی در تغییر و ارتقای سبک زندگی آنان دارد. نتایج این مطالعه می تواند گامی در جهت تدوین راهبردهای آموزشی و ارتقای سبک زندگی سالم سالمندان باشد.

Title: تأثیر هشت هفته تمرین ترکیبی آب- خشکی بر قدرت اندام تحتانی و سرعت راه رفتن مردان سالمند

http://salmandj.uswr.ac.ir/article-1-544-fa.html

اهداف: از آنجائی که ضعف قدرت و استقامت عضلات اندام تحتانی می‌تواند بر سرعت راه رفتن سالمندان اثرگذار باشد، این تحقیق تأثیر هشت هفته تمرین ترکیبی آب- خشکی بر قدرت اندام تحتانی و سرعت راه رفتن مردان سالمند را مورد مطالعه قرار داد. مواد و روش‌ها: در این مطالعه‌ نیمه تجربی 16 مرد سالمند با میانگین و انحراف سنی 2/74±62/75 سال در دو گروه کنترل (9 نفر) و تجربی (7 نفر) به عنوان آزمودنی شرکت کردند. گروه تمرین به مدت هشت هفته (دو جلسه در هفته)، در برنامه‌ تمرین استقامتی ترکیبی آب و خشکی (به‌طور متناوب یک جلسه در آب و یک جلسه در خشکی) شرکت کردند در حالی که گروه کنترل به زندگی عادی خود ادامه دادند. قبل و بعد از اعمال برنامه‌ی تمرین از هر دو گروه کنترل و تجربی آزمون برخاستن از صندلی برای سنجش قدرت عضلات اندام تحتانی و سرعت راه رفتن در مسیری 10 متری به عمل آمد. از آزمون t وابسته به منظور ارزیابی تغییرات درون گروهی و آزمون t مستقل جهت مقایسه بین دو گروه در سطح معناداری 0.05 استفاده شد. یافته‌ها: اختلاف معنی‌داری بین قدرت/استقامت اندام تحتانی، پس از اعمال دوره برنامه تمرینی بین دو گروه کنترل و تجربی وجود داشت. ضمن اینکه اختلاف معنی دار قدرت اندام تحتانی و سرعت راه رفتن قبل و بعد از تمرین در گروه تجربی مشاهده شد (P<0.05). نتیجه‌گیری: با توجه به یافته‌های تحقیق برنامه ترکیب تمرینات استقامتی آب و خشکی با هدف افزایش قدرت اندام تحتانی و بهبود سرعت راه رفتن مردان سالمند، توصیه می‌شود.

Title: بررسی ارتباط مؤلفه‌های ترکیب بدنی با تعادل ایستا، پویا و سا‌بقه زمین‌خوردن در افراد سا‌لمند فعال

http://salmandj.uswr.ac.ir/article-1-434-fa.html

اهداف: تحقیق حاضر به منظور بررسی ارتباط مولفه‌های ترکیب بدنی با تعادل ایستا، پویا و سا‌بقه زمین خوردن در افراد سا‌لمند فعال انجام شد. مواد و روش‌ها: این پژوهش، یک تحقیق ارتباط سنجی بود، که در آن 45 زن سالمند فعال، به صورت داوطلبانه مشارکت نمودند. به منظور تعیین میزان مولفه های ترکیب بدنی (توده چربی بدن، توده بدون چربی بدن، شاخص توده بدنی و نسبت دور کمر به دور باسن) آزمودنی‌ها از دستگاه Body Composition Analyzer وجهت تعیین تعادل ایستا و پویا به ترتیب از آزمون‌های ثبات وضعیتی و خطر افتادن به وسیله دستگاه تعادلی بایودکس استفاده شد. تعداد زمین خوردن افراد دریک سال گذشته ازطریق پرسش از افراد محاسبه شد. تجزیه و تحلیل آماری، با استفاده از آزمون ‎همبستگی پیرسون، در سطح معنی‌داری P≤0/05 و با نرم افزار SPSS انجام شد. یافته‌ها: نتایج پژوهش حاضر نشان داد که بین تمامی مولفه‌های ترکیب بدنی با تعادل ایستا، پویا و‌ تعداد زمین خوردن ارتباط معناداری وجود داشت (P≤0/05). نتیجه‌گیری: یافته‌های این تحقیق نشان دادند که بین همه مولفه‌های ترکیب بدنی با شاخص‌های تعادل ایستا، پویا و تعداد زمین خوردن درافراد سالمند فعال، ارتباط معناداری وجود دارد. بنابراین، به نظر می‌رسد فعالیت بدنی از طریق بهبود فاکتورهای ترکیب بدنی افراد سالمند موجب ارتقای تعادل ایستا، پویا و کاهش میزان زمین خوردن این افراد می‌گردد.
